# Identification of hub genes associated with acute kidney injury induced by renal ischemia–reperfusion injury in mice

**DOI:** 10.3389/fphys.2022.951855

**Published:** 2022-09-29

**Authors:** Sheng He, Lili He, Fangran Yan, Junda Li, Xiaoting Liao, Maoyao Ling, Ren Jing, Linghui Pan

**Affiliations:** ^1^ Department of Anesthesiology, Guangxi Medical University Cancer Hospital, Nanning, China; ^2^ Guangxi Engineering Research Center for Tissue and Organ Injury and Repair Medicine, Nanning, China; ^3^ Guangxi Key Laboratory for Basic Science and Prevention of Perioperative Organ Disfunction, Nanning, China; ^4^ Guangxi Clinical Research Center for Anesthesiology, Nanning, China; ^5^ Department of Anesthesiology, The First Affiliated Hospital of Southern China University, Hengyang, China; ^6^ Department of Anesthesiology, The Second Affiliated Hospital of Southern China University, Hengyang, China

**Keywords:** acute kidney injury, ischemia reperfusion, hub genes, Stat3, MYC

## Abstract

**Background:** Acute kidney injury (AKI) is a severe clinical syndrome, and ischemia–reperfusion injury is an important cause of acute kidney injury. The aim of the present study was to investigate the related genes and pathways in the mouse model of acute kidney injury induced by ischemia–reperfusion injury (IRI-AKI).

**Method:** Two public datasets (GSE39548 and GSE131288) originating from the NCBI Gene Expression Omnibus (GEO) database were analyzed using the R software limma package, and differentially expressed genes (DEGs) were identified. Gene Ontology (GO) and Kyoto Encyclopedia of Genomes (KEGG) and gene set enrichment analysis (GSEA) were performed using the differentially expressed genes. Furthermore, a protein-protein interaction (PPI) network was constructed to investigate hub genes, and transcription factor (TF)–hub gene and miRNA–hub gene networks were constructed. Drugs and molecular compounds that could interact with hub genes were predicted using the DGIdb.

**Result:** A total of 323 common differentially expressed genes were identified in the renal ischemia–reperfusion injury group compared with the control group. Among these, 260 differentially expressed genes were upregulated and 66 differentially expressed genes were downregulated. Gene Ontology enrichment and Kyoto Encyclopedia of Genes and Genomes analysis results showed that these common differentially expressed genes were enriched in positive regulation of cytokine production, muscle tissue development, and other biological processes, indicating that they were involved in mitogen-activated protein kinase (MAPK), PI3K-Akt, TNF, apoptosis, and Epstein–Barr virus infection signaling pathways. Protein-protein interaction analysis showed 10 hub genes, namely, *Jun*, *Stat3*, *MYC*, *Cdkn1a*, *Hif1a*, *FOS*, *Atf3*, *Mdm2*, *Egr1*, and *Ddit3*. Using the STRUST database, starBase database, and DGIdb database, it was predicted that 34 transcription factors, 161 mi-RNAs, and 299 drugs or molecular compounds might interact with hub genes.

**Conclusion:** Our findings may provide novel potential biomarkers and insights into the pathogenesis of ischemia–reperfusion injury–acute kidney injury through a comprehensive analysis of Gene Expression Omnibus data, which may provide a reliable basis for early diagnosis and treatment of ischemia–reperfusion injury–acute kidney injury.

## Introduction

Acute kidney injury (AKI) is a global public health challenge in hospitalized patients admitted to hospitals (10–15% of all hospitalizations) (Ronco et al., 2019) and in patients in the intensive care unit (ICU) where its prevalence can sometimes exceed 50% (Hoste et al., 2015). It was also linked to the increased ICU stay length and, hence, considerable healthcare resource consumption. Renal insufficiency, mesenteric vasoconstriction, infection, sepsis, and other conditions may contribute to AKI. Different causes indicate different pathogeneses. Accurate and timely identification of the cause and pathogenesis of AKI is an early basis for efficient targeted therapy in the current era of individualized therapy. Extensive investigations of AKI in clinical and basic studies have been conducted, mainly focusing on the early AKI diagnosis, injury location, etiology identification, and related mechanistic pathways (Peerapornratana et al., 2019; Jiang et al., 2021). However, biomarkers that represent renal injury, repair, and function are still being studied for improved diagnosis and treatment. For example, for AKI with insufficient volume, volume replacement is recommended, whereas for AKI with heart failure, direct treatment of heart failure is recommended without paying much attention to the increase in creatinine. Hypoxia may be the only connection between these two causes. Currently, specific biomarkers of renal hypoxia in urine or plasma are not commonly used in the clinic (Endre and Mehta, 2020).

Improving Global Outcomes Clinical Practice Guidelines define AKI as an elevation in serum creatinine greater than 0.3 mg/dl above baseline or prolonged oliguria (greater than 6 h) (Kellum et al., 2013), and both have several limitations. Acute changes in the glomerular filtration rate (GFR) are not consistent with changes in serum creatinine levels, as the balance between production and elimination takes days to occur. Therefore, serum creatinine underestimates the extent of renal function loss, especially in the first 48 h after injury and tends to increase when a substantial amount (∼50%) of GFR is lost (Kellum et al., 2021). Furthermore, serum creatinine levels are not only affected by GFR but also by age, sex, muscle mass, muscle metabolism, medication, and hydration status, further delaying the diagnosis and treatment.

In recent years, much of the research on nephrology has focused on identifying AKI biomarkers to address the limitations of these traditional diagnostic indicators. Neutrophil gelatinase-associated lipocalin (NGAL) is the most widely studied biomarker for AKI. NGAL has been found to be the most useful biomarker after cardiac surgery (especially in children) and kidney transplants and in critically ill patients (Khawaja et al., 2019; Robertson et al., 2019; Marcello, 2022). It is currently used to predict and diagnose AKI, as well as to predict short- and long-term outcomes and decision-making regarding AKI treatment (such as renal replacement therapy (RRT)). Tiranathanagul et al. (2013) performed a study to determine the most appropriate cut-off value to predict the initiation of RRT. A urine NGAL level of 2000 ng/ml and plasma NGAL level of 1,000 ng/ml could predict AKI requiring RRT with AUCs of 0.81 for both, respectively. However, the expression of NGAL still lacks specificity for AKI, and the diversity of test kits in the market means that the cut-off value is not clear. The liver-type fatty acid-binding protein (L-FABP) is often used to predict AKI. L-FABP exists not only in the liver but also in many organs, such as intestines, stomach, lungs, and kidneys.

L-FABP can be detected in urine and is linearly associated with hypoperfusion markers (lactic acid) in a study of 249 patients with severe disease. This is considered an obvious indicator of renal interstitial hypoxia. In critically ill patients, compared with other biomarkers, including NGAL, IL-18, and albumin, L-FABP has more advantages in predicting AKI (Srisawat and Kellum, 2020). Recently, urinary L-FABP has been approved as a biomarker of AKI in Japan. However, few studies have explored the role of L-FABP in predicting short- or long-term renal outcomes or mortality, and there is no standard tipping point for AKI diagnosis. Insulin-like growth factor-binding protein-7 (IGFBP-7) and tissue inhibitor of metalloprotease-2 (TIMP-2) have been implicated in the early stage of cellular stress in G1 cell cycle arrest by blocking the effect of cyclin-dependent protein kinase complexes (Wang et al., 2017). The scores of IGFBP-7 × TIMP-2 can be used to monitor the incidence and recovery of moderate to severe AKI in patients undergoing cardiac surgery with cardiopulmonary bypass (CPB) (Jia et al., 2022; Tao et al., 2022). A study showed that urinary IGFBP-7 × TIMP-2 at 2 h after CPB initiation showed an AUC of 0.76 for predicting the severity of AKI. However, another study showed a poor predictive value for low-risk inpatients in non-intensive care units (ICUs), with a specificity of only 46% (Bihorac et al., 2014).

Thus, the current approach strives to find novel AKI biomarkers for recognition of AKI in its early stages, and early response is geared to prevent progression to more severe stages. Novel biomarkers may also be used to probe into the underlying mechanisms leading to renal damage, and recovery in patients is essential.

Recent multiple experimental analyses indicated that Rplp1 significantly upregulated display dramatically increased in the kidneys of Cis-AKI and display dramatically increased after 3 days and decreased after 7 days (Lin et al., 2021). Moreover, a previous study focused on the relationship between the expression of TIMP1 and the incidence of sepsis-associated acute kidney injury (SAAKI). Tang et al. (2021) found that the expression of serum TIMP1 was much higher in patients with SAAKI than in those without SAAKI and in the control group. An increasing number of studies have been performed on AKI gene expression profiles. However, most studies have focused on Cis-AKI and SAAKI, which is why the underlying mechanisms of AKI in ischemia–reperfusion injury (IRI)-induced acute kidney injury (IRI-AKI) remain largely unclear. Another bioinformatics analysis based on the GEO database identified several key genes associated with AKI, such as *Suchvcr1*, *Krt20*, *Sox9*, *Egr1*, and *Timp1* (Chen et al., 2020). However, no more in-depth studies were conducted, i.e., there are no studies of miRNA associated with differential gene regulation and small molecules interacting with key genes, which lacks theoretical support for clinical treatment. Microarrays and high-throughput sequencing have been widely used to explore the molecular mechanisms of a series of AKI (Deng et al., 2021; Tang et al., 2021; Wang et al., 2021). However, the results of these studies are inconsistent, including differentially expressed genes (DEGs) and related pathways, which may be related to the heterogeneity of each experiment. Thus, single-cohort studies still have limitations that cannot be ignored. Integrated analysis of data from different gene expression profiles may be more reliable, and more reliable and effective diagnostic biomarkers and therapeutic targets may be identified.

The present study aimed to gain an in-depth understanding of its mechanism and identify potential molecular markers for prognosis and early detection, as well as drug targets for the treatment of AKI. Two datasets [GSE39548 (Correa-Costa et al., 2012) and GSE131288 (Aufhauser et al., 2021)] were downloaded and analyzed from the Gene Expression Omnibus (GEO) database to identify genes that are differentially expressed (DEGs) in AKI caused by IRI. Functional enrichment analysis and protein-protein interaction (PPI) network construction were performed for the targeted genes. Differentially expressed miRNAs were screened, followed by the predation of targeted miRNAs and the construction of miRNA–hub gene networks. Finally, we used the Drug–Gene Interaction Database (GGIdb database) to predict drug molecules that could interact with hub genes to identify potential drugs for the treatment of AKI. Our study provides new insights into the molecular mechanisms underlying AKI based on its pathophysiology, which could be further utilized to explore novel diagnostic and therapeutic strategies.

## Materials and methods

### Data preprocessing

In order to identify potential molecular markers in IRI-AKI, we used the keywords “IRI, AKI” to search on the GEO database (https://www.ncbi.nlm.nih.gov/geo/), and the mRNA expression profiles of two datasets were selected for the present study. Data GSE39548 (Correa-Costa et al., 2012) used the GPL7202 platform (Agilent-014868 Whole Mouse Genome Microarray) which included four IRI-AKI and four control samples. Data GSE131288 (Aufhauser et al., 2021) used the GPL16570 platform (Affymetrix Mouse Gene 2.0 ST Array) which included three IRI-AKI and three control samples. GEO databases were provided as the raw data. Subsequently, the gene expression profile data of the two datasets were obtained using filtered preprocessing, which included the background correction and quantile normalization.

### Identification of differentially expressed genes

The classical Bayesian algorithm in the “limma” package (Ritchie et al., 2015) was used to correctly identify differentially expressed genes (DEGs) in IRI-AKI samples compared with the non-injured renal samples in two datasets (GSE39548 and GES131288). Criteria for the statistical significance difference of DEGs were | log2 fold change (FC) | ≥ 1 in expression and an adjusted *p*-value (false discovery rate, FDR) < 0.05. Volcano plots of the expression of all DEGs were generated using the ggplot2 package in R, and heatmaps were generated using the pheatmap package (Khomtchouk et al., 2014). The Venn diagram was drawn using the ggplot2 package, and the intersection of DEGs of the two datasets was used to obtain the genes with common differential expression.

### Gene Ontology and Kyoto Encyclopedia of Genes and Genomes pathway enrichment analyses of differentially expressed genes

Gene Ontology (GO; http://geneontology.org) provides structured, computable knowledge regarding the functions of genes for identifying molecular function (MF), biological process (BP), and cellular component (CC) attributes for high-throughput genome or transcriptome data (The Gene Ontology, 2019). The Kyoto Encyclopedia of Genes and Genomes (KEGG; https://www.kegg.jp/) is a manually curated resource that integrates 18 databases categorized into systems, genomic, chemical, and health information, for systematic analysis of gene functions and associating related gene sets with their pathways (Kanehisa et al., 2021). GO annotation and KEGG pathway enrichment analyses were conducted for DEGs in R using the clusterProfiler package (Yu et al., 2012). The adjusted *p* < 0.05 and count number of enriched genes more than five were the threshold for screening the main enrichment functions and pathways of differential genes.

### Gene set enrichment analysis (GSEA)

In this study, gene set enrichment analysis (GSEA), performed using the clusterProfiler (3.14) R package (Wu et al., 2021), was used to elucidate the significant function and pathway differences between the IRI-AKI groups and control groups. The number of gene set permutations was 1,000 times for each analysis. Pathways enriched for each phenotype were obtained from the CP gene sets of the C2 subset (c2. cp.all.v7.0. symbols.gmt) as the preset gene sets for enrichment analysis and classified by the adjusted *p*-value (<0.05), FDR q-value (<0.25), and normalized enrichment score (|NES| > 1).

### Protein–protein interaction network construction and identification of hub genes

To further explore the interaction among the common differentially expressed genes (co-DEGs), a protein–protein interaction network (PPI) of co-DEGs was identified using the Search Tool for the Retrieval of Interacting Genes (STRING) (http://string-db.org/) database (Szklarczyk et al., 2019), which is an online biological resource database. This database has a comprehensive score for each PPI relationship pair that is distributed between 0 and 1; the higher the total score, the more reliable is the PPI relationship. The commonly used combined score threshold is 0.4. In this study, an interaction with a combined score >0.4 was considered statistically significant. The PPI network was visualized using Cytoscape software (Kohl et al., 2011), and its plug-in NetworkAnalyzer was used to analyze the relevant properties of the nodes in the network. cytoHubba was used to identify the hub genes in the PPI network of co-DEGs, and the shade of color corresponds to its criticality.

### Transcription factor (TF)–hub gene network and miRNA–hub gene network construction

TRRUST (https://www.grnpedia.org/trrust/) is a reliable and intuitive tool for human and mouse transcriptional regulatory networks (Han et al., 2018). Containing 8444 TF-target regulatory relationships of 800 human TFs, the TRRUST database can provide the key TFs for multiple genes and information on how these interactions are regulated. We predicted the TFs capable of regulating hub genes using the STRUST database. The starBase V2.0 database (http://starbase.sysu.edu.cn/) contains data from five databases: TargetScan, PicTar, PITA, miRanda/mirSVR, and RNA22 which constructed the most comprehensive miRNA–lncRNA, miRNA–pseudogene, miRNA–circRNA, miRNA–mRNA, and protein–RNA interaction networks (Li et al., 2014). We used the starBase database to predict the miRNAs that can target and bind to the hub genes. The screening standard was that there were predicted results in at least three databases. Finally, we used Cytoscape software (Shannon et al., 2003) to map the two regulatory networks of TF–hub genes and miRNA–hub genes.

### Identification of potential drugs

The Drug–Gene Interaction Database (DGIdb, www.dgidb.org) is a web resource that provides information on drug–gene interactions and druggable genes from publications, databases, and other web-based sources and can be used to identify drugs that interact with these genes (Freshour et al., 2021). We used the DGIdb to predict the drugs and molecular compounds that can interact with the hub genes. The drug–hub gene interaction network was plotted using Cytoscape software (Shannon et al., 2003).

## Results

### Identification of differentially expressed genes

**FIGURE 1:**
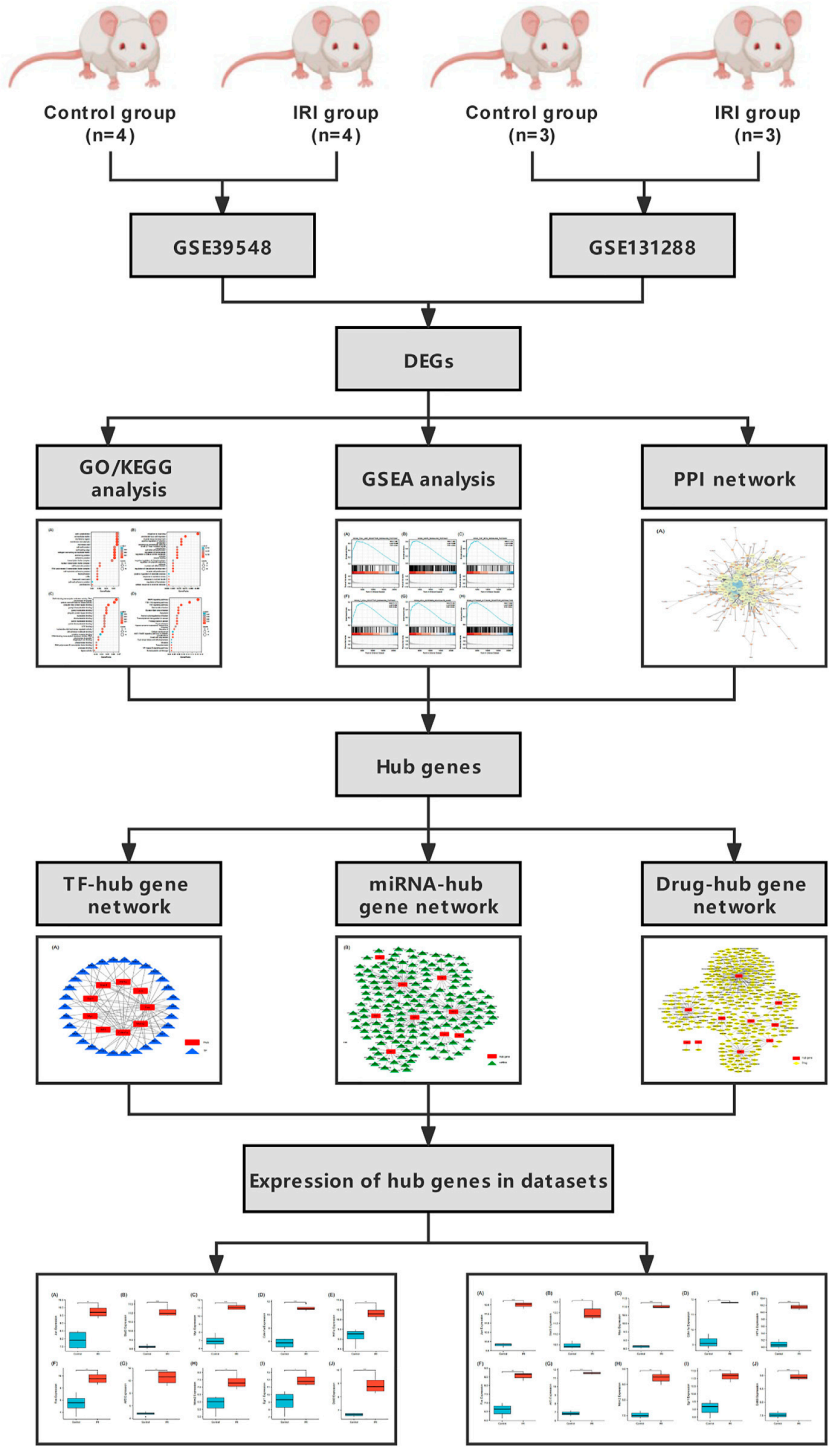
Flowchart of this study.

**FIGURE 2:**
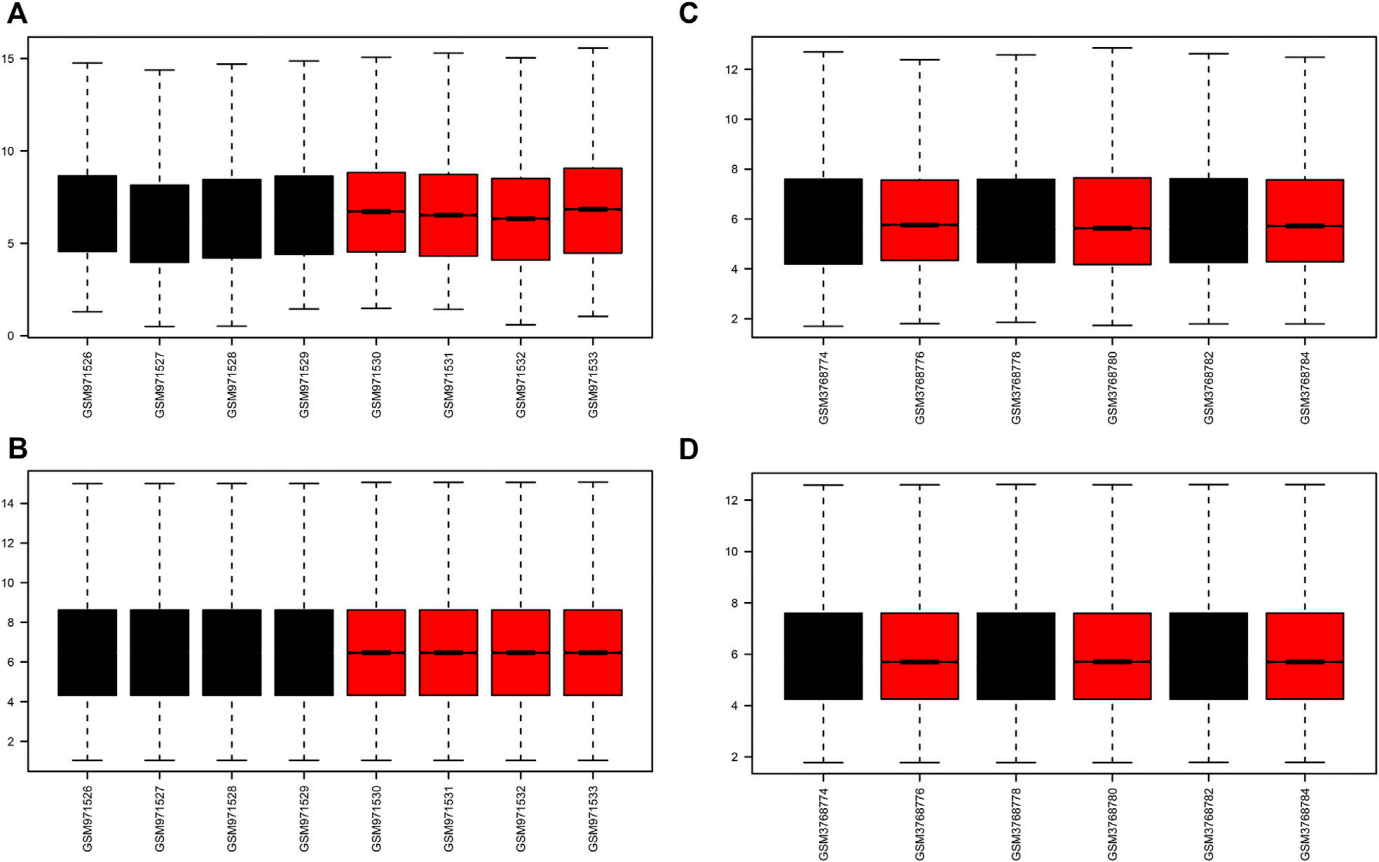
Boxplots of GEO dataset samples before and after correction. **(A–B)** GSE39548 dataset before and after correction; **(C–D)** GSE131288 dataset before and after correction.

**FIGURE 3:**
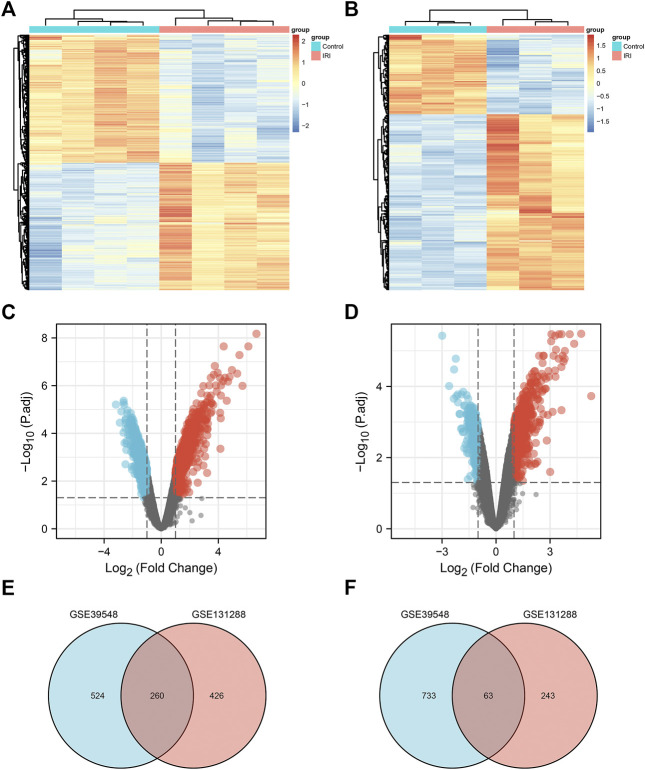
Differentially expressed genes. **(A)** Heatmap of the GSE39548 dataset; **(B)** heatmap of the GSE131288 dataset; **(C)** volcano map of the GSE39548 dataset; **(D)** volcano map of the GSE131288 dataset; **(E)** Venn diagram of the intersection of upregulated differentially expressed genes in GSE39548 and GSE131288;**(F)** intersection of downregulated differentially genes in the two datasets.

A flow chart of the study is shown in Figure 1. Before the next analysis, we performed quality control for the two datasets (GSE39548 and GES131288). The boxplot results before and after normalization are shown in Figure 2. The gene expression in each sample of the two datasets was uniform after normalization, and the qualified data were reliable and could be used for further analyses. Based on the cutoff criteria (adjust *p*-value < 0.05 and |logFC| >2), a total of 1,580 DEGs were screened from the GSE39548 dataset, of which 784 were upregulated and 796 were downregulated in the IRI group. In the GSE131288 dataset, a total of 992 DEGs were screened, among which 686 were upregulated in the IRI group and 306 were downregulated in the IRI group. A heatmap was constructed to visually present the expression change of the upregulated and downregulated DEGs in each dataset and reveal the distribution of the gene expression data of each subset (Figures 3A,C), which are marked in different colors in the volcano map (Figures 3B,D). To further explain our data, the upregulated and downregulated DEGs of GSE39548 and GSE131288 expressed in the heatmap are shown in our Supplementary Tables S1, S2, and the DEGs expressed in the volcano map are shown in the Supplementary Tables S3, S4, In addition, after taking the intersection of DEGs of the two datasets, 323 genes with common DEGs (co-DEGs) were obtained, of which 260 were upregulated and 63 downregulated in the IRI group, which were visualized by the Venn diagram (Figures 3E,F); Venn diagrams for upregulated and downregulated DEGs are shown in Supplementary Tables S5–S6.

We performed differential expression analysis through GSE39548 and GSE131288 datasets to obtain differentially expressed genes (DEGs) and then performed GO/KEGG/GSEA enrichment analysis, respectively. We screened hub genes through PPI interaction network analysis. Then, hub genes were used to predict transcription factors, miRNA, and drug small molecules, and the differences in the expression of Hub genes were verified in the two datasets.

### GO and KEGG pathway enrichment analyses of 323 DEGs

**FIGURE 4:**
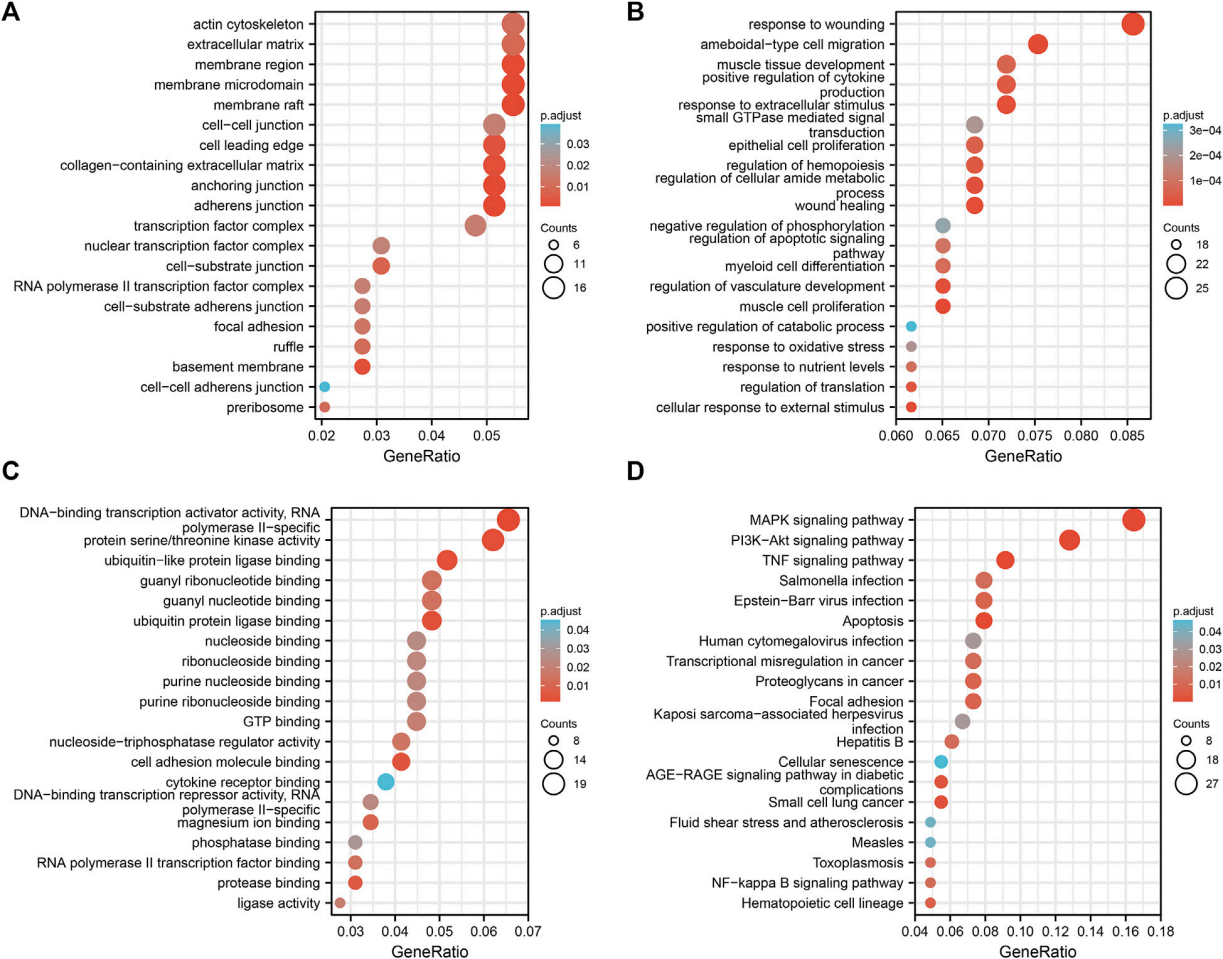
GO/KEGG enrichment analyses of common differential genes.

The DAVID database was used to determine the potential functions of DEGs and identify the overrepresented GO categories in the biological processes of these 323 DEGs. As shown in Figures 4A–C and Supplementary Table S1, for biological process (BP), DEGs were mainly enriched in cell components such as membrane raft, membrane microdomain, membrane region, extracellular matrix, and actin cytoskeleton component (CC), response to ritz, ameboidal-type cell migration, response to extracellular stimulus, positive regulation of cytokine production, and muscle tissue development); for molecular function (MF), DEGs were mainly enriched in cell components such as DNA-binding transcription activator activity, RNA polymerase II-specific, protein serine/threonine kinase activity, ubiquitin-like protein ligase binding, ubiquitin protein ligase binding, and guanyl nucleotide binding. KEGG pathway analysis was conducted to ascertain which 323 common DEGs participated. A total of 20 significantly enriched pathways were identified with an adjusted *p*-value of <0.05, and 20 significantly enriched pathways were obtained. DEGs were mainly enriched in the mitogen-activated protein kinase (MAPK), phosphatidylinositol 3 kinase/protein kinase B (PI3K-Akt), tumor necrosis factor (TNF), apoptosis, and Epstein–Barr virus infection signaling pathways (Figure 4D and Supplementary Table S2).

### Gene set enrichment analysis (GSEA)

**FIGURE 5:**
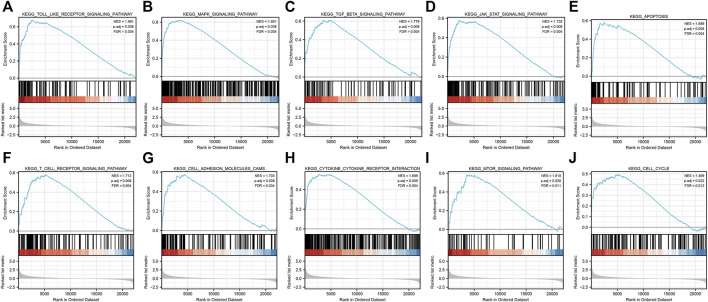
GSEA of common differential genes. **(A)** Toll-like receptor signaling pathway, **(B)** MAPK signaling pathway, **(C)**TGF-β signaling pathway, **(D)** JAK-STAT signaling pathway, **(E)** apoptosis signaling pathway, **(F)** T-cell receptor signaling pathway, **(G)** cell adhesion molecules (CAMs) signaling pathway, **(H)**cytokine–cytokine receptor interaction signaling pathway, **(I)** mTOR signaling pathway, and **(J)** cell cycle signaling pathway. Note: all these pathways were mainly enriched in AKI caused by ischemia–reperfusion.

**FIGURE 6:**
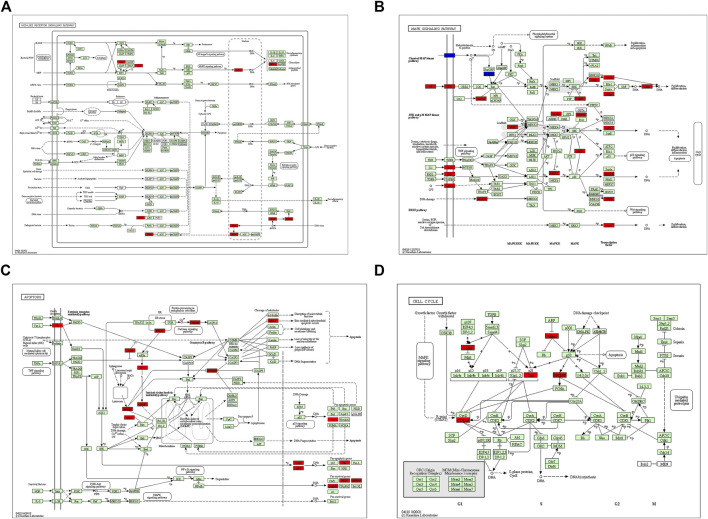
KEGG pathway enriched by common differential genes. **(A)** NOD-like receptor signaling pathway, **(B)** MAPK signaling pathway, **(C)** apoptosis signaling pathway, and **(D)** cell cycle signaling pathway. Note: genes marked in red in signaling pathways are expressed as differentially expressed genes in this study.

After GSEA which used the CP gene sets of subsets of C2 as the predefined gene set, we found that the NOD-like receptor signaling pathway, Toll-like receptor signaling pathway, MAPK signaling pathway, TGF-β signaling pathway, JAK-STAT signaling pathway, apoptosis signaling pathway, T-cell receptor signaling pathway, cell adhesion molecules (CAMs), cytokine–cytokine receptor interaction, and cell cycle were positively correlated with IRI gene expression patterns (Figure 5 and Supplementary Table S3). In addition, we plotted four signaling pathways associated with ischemia–reperfusion injury (Figure 6), including the NOD-like receptor signaling pathway, MAPK signaling pathway, apoptosis signaling pathway, and cell cycle signaling pathway, using the KEGG-MAP database.

### Protein–protein interaction network construction and identification of hub genes

**FIGURE 7:**
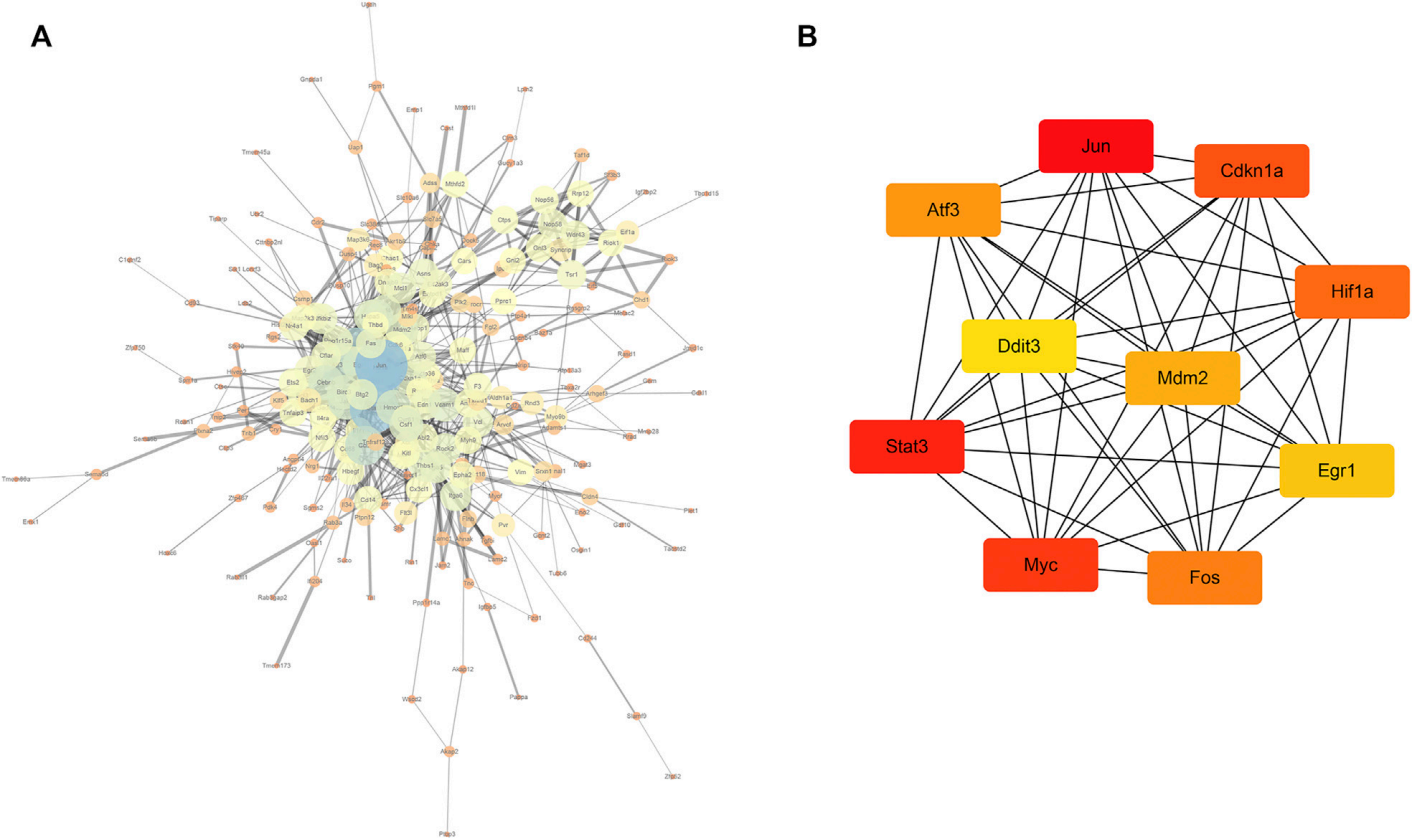
PPI network and core gene screening **(A)** using the NetworkAnalyzer plug-in to modify the PPI network; the larger the node, the greater the degree of connectivity (degree), and the thicker the line, the greater the combined_score value; **(B)** top 10 core gene interaction networks; the darker the color, the more powerful the critical degree.

In total, 323 common differentially expressed genes (co-DEGs) obtained from the aforementioned analysis were input into the STRING database to screen the proteins interacting with them, and the obtained results were imported into Cytoscape software to build a PPI network. The NetworkAnalyzer plug-in was used to calculate the directionless scores for each node in the PPI network. The degree value of each node was obtained. The node size and color represents the degree value, and the edge thickness represents the edge-combined score value (Figure 7A). In addition, the top 10 genes were identified as core genes by calculating the tightness of the connection of the node using the cytoHubba plug-in, namely, *Jun*, *Stat3*, *Myc*, *Cdkn1a*, *Hif1a*, *Fos*, *Atf3*, *Mdm2*, *Egr1*, and *Ddit3* (Figure 7B). There were strong interactions among these hub genes, which may have an effect on the pathophysiological process of AKI.

### Transcription factor–hub gene network and miRNA–hub gene network construction

**FIGURE 8:**
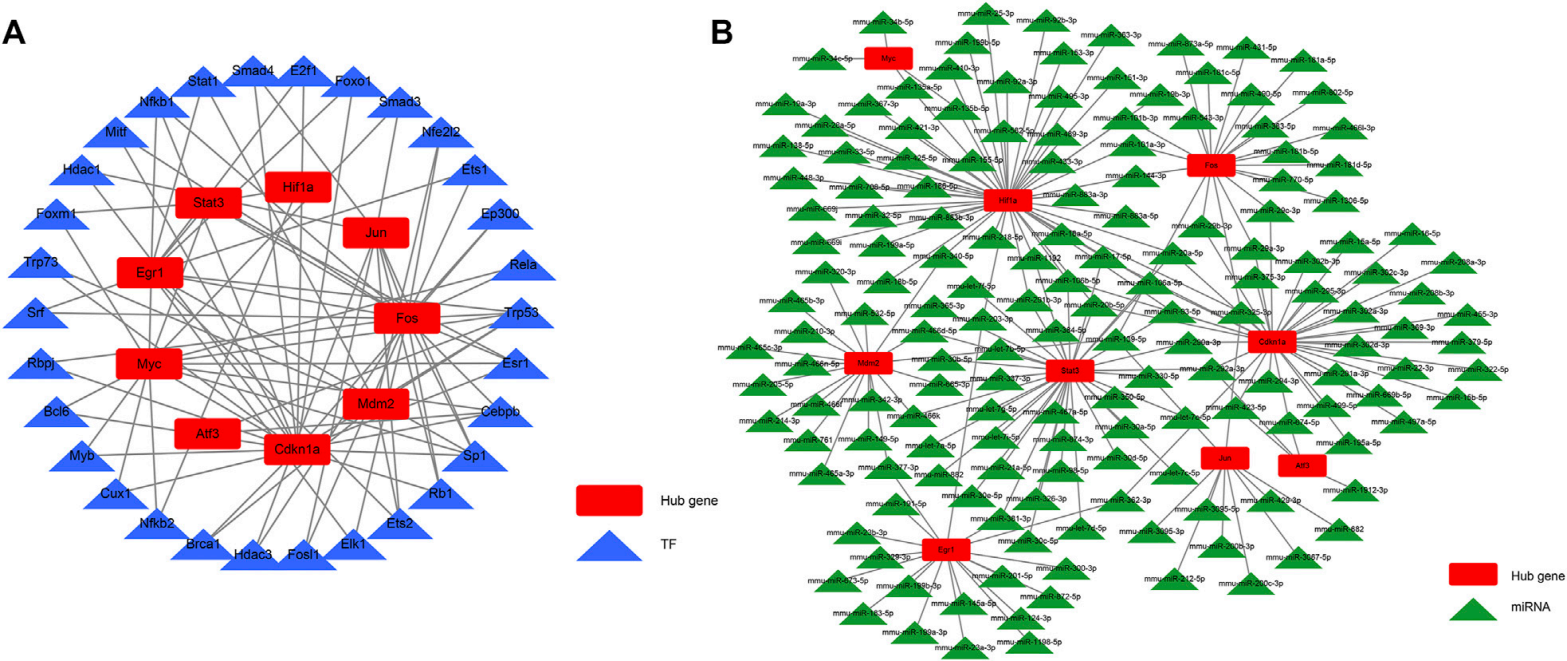
TF–hub gene interaction network and miRNA–hub gene interaction network. **(A)** TF–hub gene network; **(B)** miRNA–hub gene network.

We aimed to better understand the regulatory roles of hub genes in the pathogenesis of AKI. We predicted 34 TFs capable of interacting with 10 hub genes using the STRUST database and built a TF–hub gene regulatory network (Figure 8A). We also predicted miRNAs that could interact with 10 hub genes through the starBase database and constructed an miRNA–hub gene interaction network (Figure 8B), and 161 miRNA–hub gene interaction networks were constructed. It can be noted that the 10 hub genes we screened had binding sites for multiple TFs and miRNAs, which can guide us to further mechanistic research.

### Identification of potential drugs that interact with hub genes

**FIGURE 9:**
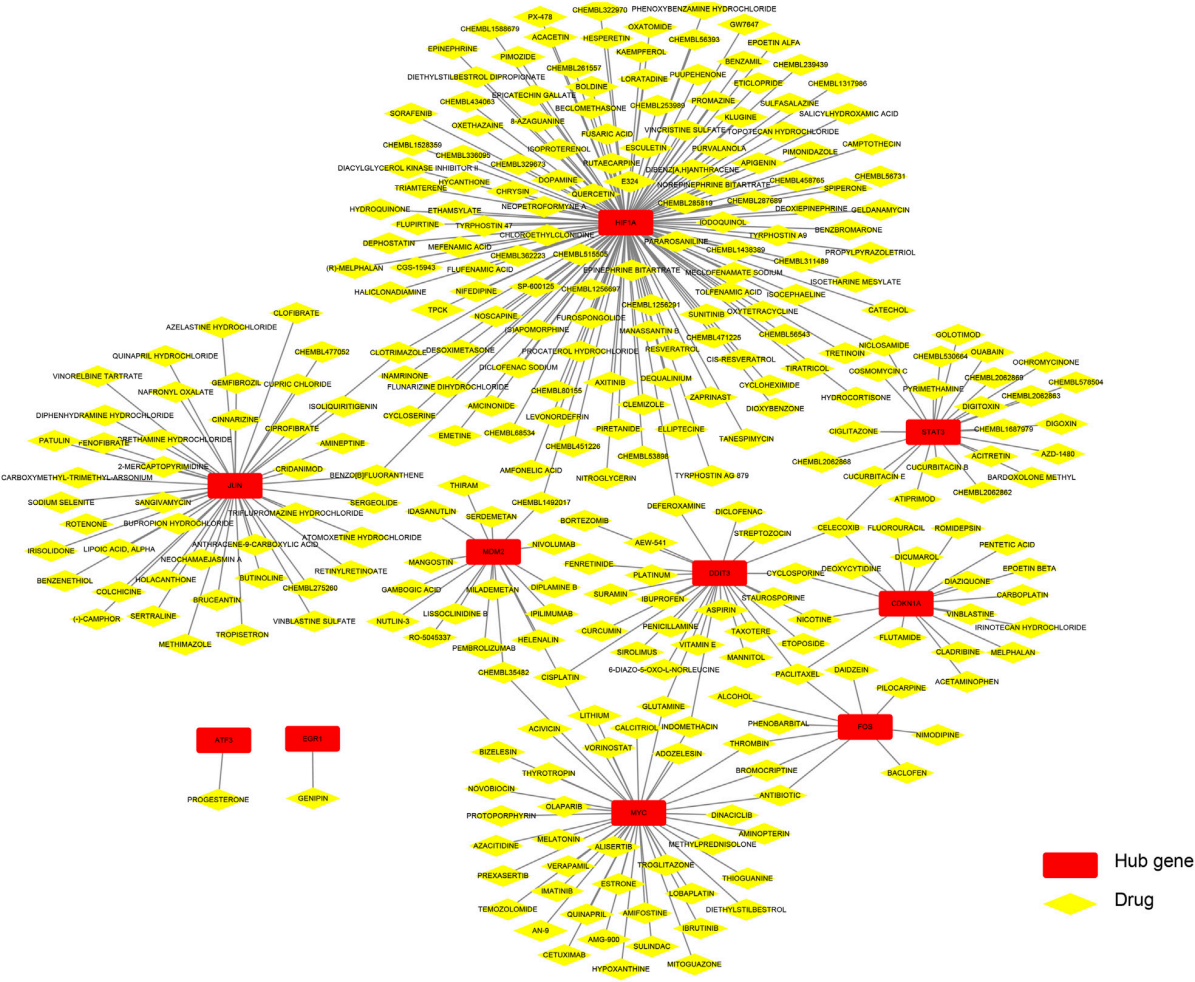
Construction of the drug–hub gene interaction network.

Figure 9 shows that drugs or molecular compounds that may interact with hub genes were predicted by the DGIdb database, and a total of 299 drugs or molecular compounds that may have regulatory relationships with hub genes were screened, among which the drugs that interact with HIF1A were the most in number.

### Expression level of core genes in IRI-AKI disease

**FIGURE 10:**
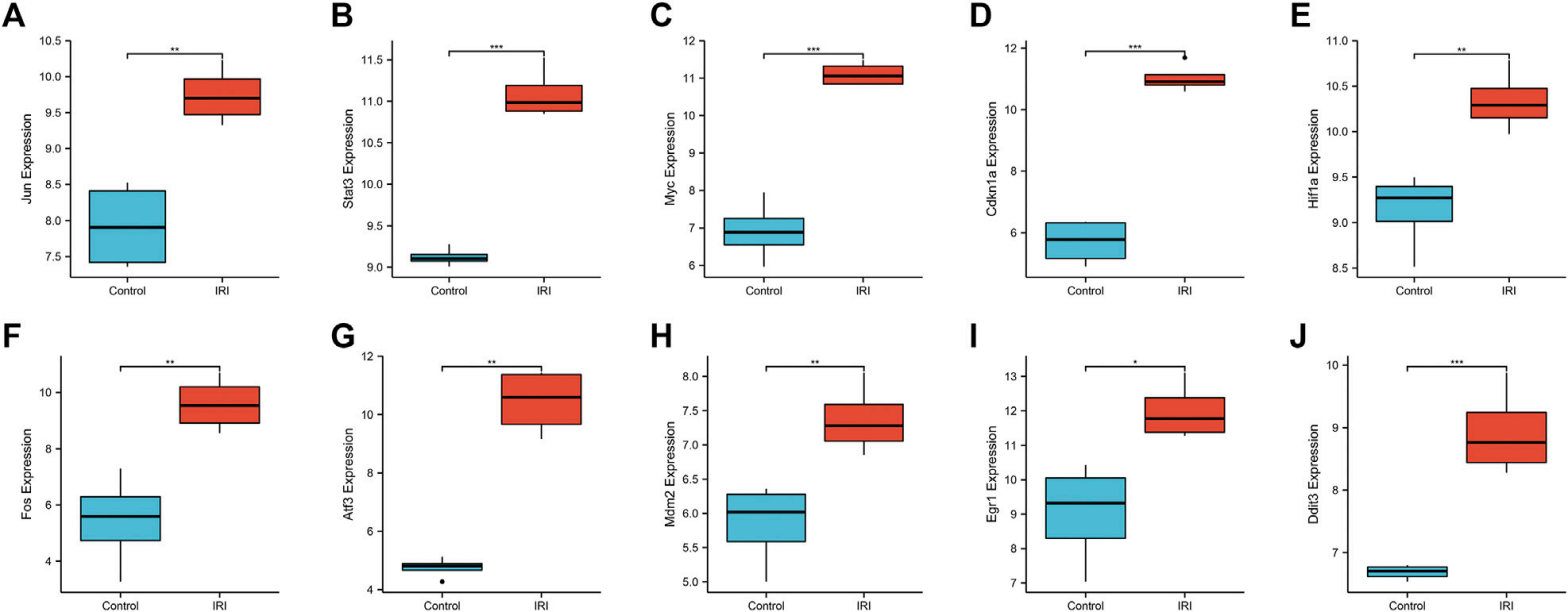
Expression levels of 10 hub genes in the GSE39548 dataset. *Jun* **(A)**, *Stat3* **(B)**, *Myc* **(C)**, *Cdkn1a* **(D)**, *Hif1a* **(E)**, *Fos* **(F)**, *Atf3* **(G)**, *Mdm2* **(H)**, *Egr1* **(I)**, and *Ddit3* **(J)**. Differential expression analysis in the IRI-AKI group vs. control group in the GSE39548 dataset. Note: *, *p* < 0.05; **, *p* < 0.01; ***, *p* < 0.001.

**FIGURE 11:**
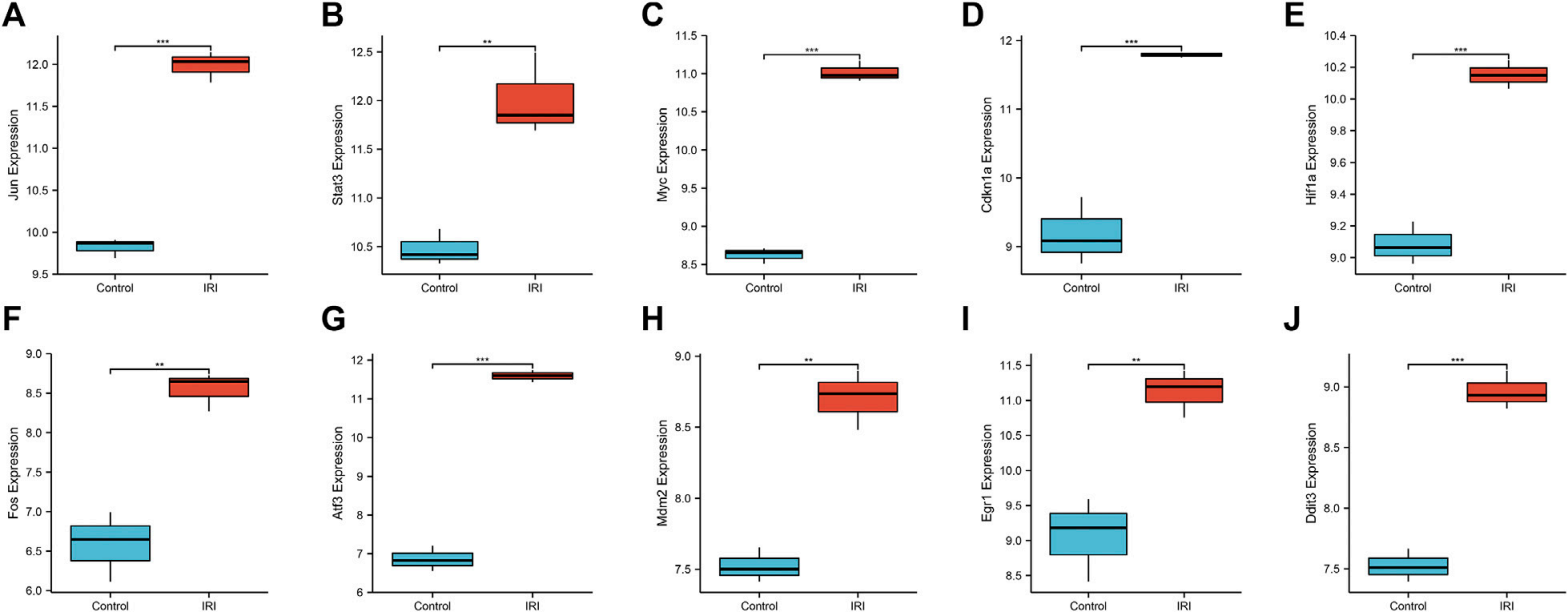
Expression levels of 10 hub genes in the GSE131288 dataset. *Jun* **(A)**, *Stat3* **(B)**, *Myc* **(C)**, *Cdkn1a* **(D)**, *Hif1a* **(E)**, *Fos* **(F)**, *Atf3* **(G)**, *Mdm2* **(H)**, *Egr1* **(I)**, and *Ddit3* **(J)**. Differential expression analysis in the IRI-AKI group vs. control group in the GSE131288 dataset. Note: *, *p* < 0.05; **, *p* < 0.01; ***, *p* < 0.001.

We used a boxplot to show the expression levels of the 10 hub genes in IRI-AKI mouse kidney tissue. In the GSE39548 dataset, 10 hub genes were significantly more expressed in the IRI-AKI group than in the control group (Figure 10). Similarly, in the GSE131288 dataset, 10 hub genes were significantly more expressed in the IRI-AKI group, with statistically significant differences (Figure 11).

## Discussion

AKI is a global public health concern associated with high morbidity, mortality, and healthcare costs (Ronco et al., 2019), and early diagnosis, appropriate classification, and timely treatments in the initial stage of AKI play a crucial role in reducing mortality. However, its pathogenesis is extremely complex and not fully understood. Bioinformatics analyses have enabled us to understand the molecular mechanisms of AKI occurrence and development and have been used to explore potential targets for AKI diagnosis and treatment. Previous microarray studies have often focused on the role of a single molecule and are largely based on a sepsis-induced AKI model (Tang et al., 2021; Ji et al., 2022). However, the vast majority of AKI in clinical work is associated with ischemic–reperfusion injury, and there have been some studies on the gene expression of IRI-AKI (Wu et al., 2016; Wu et al., 2019). Regardless, at present, there is still not enough clarity on its potential pathogenesis and related targets, and there is a lack of clear biomarkers to determine its diagnosis, development, and eventual outcome. To date, there has been no timely and accurate diagnosis or effective treatment for IRI-AKI.

Hence, we aimed to identify the novel key genes that are involved in the pathogenesis of IRI-AKI. We performed a systematic analysis of two expression profiles from the GEO database using bioinformatics analysis. In total, 323 co-DEGs were identified in the IRI-AKI group, including 260 upregulated and 63 downregulated genes. In the GO and KEGG analyses, we found that the most enriched BP and MF terms were mostly associated with the response to wounding, and DNA-binding transcription activator activity, MAPK signaling pathway, and PI3K-Akt signaling pathway were related to their mechanism of occurrence. Moreover, GSEA suggested that the MAPK pathway was the most closely correlated with the gene expression patterns. Furthermore, 10 hub genes were extracted from the PPI network of the co-DEGs, and we predicted the TFs, mi-RNAs, and small molecules of the drug interacting with 10 hub genes. Finally, we showed through the boxplot view that the expression of ten hub genes in different groups proved the accuracy of our analysis.

Signal transducer and activator of transcription 3 (STAT3) belongs to a protein family composed of seven members (STAT 1, 2, 3, 4, 5a, 5b, and 6) (Rah et al., 2022). STAT3 proteins are key downstream TFs induced by IL-6 cytokines involved in the pathogenesis of kidney disease (Dube et al., 2017; Zhou et al., 2019) that can be activated by binding to phosphorylated tyrosine residues on the cytoplasmic tails of activated cytokine receptors. STAT3 is closely related to the regulation of multiple biological pathways such as proliferation, survival, angiogenesis, and apoptosis. Park et al. (2022) found that STAT3 expression in IRI-AKI murine kidneys was significantly increased compared with that in the normal group and was closely related to the degree of kidney injury in mice. It can downregulate inflammation through the JAK2 pathway to improve renal tubule injury and inflammatory cell infiltration and reduce mortality in mice with renal IRI. In a hypoxia-induced human cell injury model using primary cultured human tubular epithelial cells (TECs), Stat3 significantly inhibited the inflammatory response and decreased the expression of P53, which is a signature protein representing apoptosis. In addition, the expression of pSTAT3 increases in human renal tubulointerstitial and glomerular areas, and with the progression of the disease, the level of pSTAT3 expression increases accordingly in patients who transitioned from AKI to chronic kidney disease (CKD). A recent study on COVID-19-induced AKI pathways may contribute to kidney injury in some patients with COVID-19 (Salem et al., 2022). The STAT3 inhibitor Stattic^®^ treatment attenuated IRI-induced tubular damage and inflammatory cytokine/chemokine expression while decreasing macrophage infiltration and fibrosis in mouse unilateral IRI models. Similarly, *in vitro* STAT3 inhibition downregulated fibrosis and apoptosis in human tubular epithelial cells (TECs) exposed to hypoxia for 72 h and reduced the expression of inflammatory cytokines IL-6 and IL-8. STAT3 activation is associated with IRI progression and may be a significant contributor (Park et al., 2022). Hence, here, we suggested a novel strategy for AKI management using STAT3 inhibitors as the inhibition of STAT3 signaling alleviated the progression of acute kidney injury to chronic kidney disease through anti-apoptosis. In line with previous studies, our study also revealed that STAT3 is associated with the IRI-AKI progression and serves as an early diagnostic marker and therapeutic target of IRI-AKI. In line with previous studies, our research also revealed that STAT3 is associated with progression and an early diagnostic marker and therapeutic target of IRI-AKI.

Myc belongs to the superfamily of basic helix–loop–helix leucine zipper (bHLHLZ) DNA-binding proteins. It is a major TF (Beaulieu et al., 2020). Myc is believed to regulate more than 15% of human genes and is sometimes described as a “master gene regulator.” It is involved in the regulation of several cellular processes, including cell growth, cell cycle, differentiation, apoptosis, angiogenesis, DNA repair, and stem cell formation (Carroll et al., 2018; Beaulieu et al., 2020). The Myc family comprises three members: C-Myc, L-Myc, and N-Myc. Overexpression of c-Myc leads to cell proliferation and may be involved in the regulation of renal tubular cell death in AKI (Bao et al., 2014). Recent studies have also shown that Myc activation plays an important role in renal fibrosis and in the progression of AKI to CKD (Hultström et al., 2018). Myc expression is positively correlated with collagen I, a major component of the extracellular matrix, and is a well-established indicator of fibrogenesis. In the advanced stage of AKI, Myc is distinctly highly expressed. Oral administration of an Myc inhibitor can significantly reduce KIM-1 expression, which is a sensitive indicator of renal tubular injury, the accumulation of inflammation in mice, and fibrosis, while ultimately slowing the progression of AKI to CKD (Lemos et al., 2018). However, in a study by Wang et al. (2021), Myc was screened as hub genes in AKI damage caused by CIS but not in I/R injury. This is inconsistent with our conclusions, possibly due to the different animal models used in different studies and inconsistent ischemia–reperfusion times. In addition, Liu et al. (2021) found that Myc promotes MEG3 transcription in HK-2 cells after IRI. Mitophagy was activated and apoptosis was promoted, aggravating acute kidney injury caused by IRI and triggering the Wnt/β-catenin pathway by promoting PTKN overexpression. In our study, Myc was also found to be a hub gene of IRI-AKI, which is closely related to other co-DEGs, and may provide a novel theory and target for the treatment of IRI-AKI in the future.

Fos is a subunit of the activator protein-1 (AP-1) TF. AP-1 consists of various combinations of Fos (c-Fos, FosB, Fra-1, and Fra-2) and Jun (c-Jun, Jun B, and Jun D) proteins (Rodríguez-Berdini and Caputto, 2019). They all belong to the basic leucine zipper TF superfamily and bind the target DNA double-stranded sites with homodimer or heterodimer (Glover and Harrison, 1995). Fos has been shown to be involved in various cellular activities, such as proliferation, differentiation, survival, metabolism, hypoxia, angiogenesis, sterogenesis, and prostaglandin production (Li et al., 2016; Choi et al., 2021). Multiple studies have demonstrated that the expression of Fos in the AKI group is significantly higher than that in the control group (Zhang et al., 2019; Xu and Xu, 2022). Furthermore, the PPI network analysis showed that Fos was the hub gene with the highest degree of tight connection. Fos directly interferes with the transcription of inflammatory cytokines, such as TNF-α, IL-6, and IL-1β, and induces their high expression to boost the occurrence of AKI by binding to promoters of inflammatory cytokines (Zhang et al., 2019). In addition to its high expression in animal AKI models, Fos expression has also been demonstrated to be upregulated in severely damaged human kidney tissues compared to that in normal kidney tissues. It was further found that Fos in the IR group was also upregulated in HK-2 cells *in vitro* (Ke et al., 2021). The conclusions of these studies all support our current research that Fos is more likely to be involved in biological regulation and interconnected with IRI-AKI and could act as a biomarker, which might be used to assess the severity of IRI and verify the effectiveness of treatments.

Renal IR is a common cause of AKI due to an imbalance in tissue oxygen supply and demand, which can lead to the overproduction of reactive oxygen species and inflammatory mediators and directly or indirectly activate the apoptotic pathway (Han and Lee, 2019). Specifically, IR injury leads to the death of renal tubular epithelial cells and eventually leads to an irreversible loss of renal function. The mitogen-activated protein kinase (MAPK) cascade is a key signaling pathway that regulates a variety of cell biological processes such as proliferation, differentiation, apoptosis, and stress response under normal and pathological conditions (Guo et al., 2020). Sun et al. (2022) found that in mice with acute kidney injury induced by ischemia–reperfusion, various proinflammatory cytokines (such as TNF-α and IL-1β) were released in the kidney tissue, which promoted kidney injury; phosphorylation of MAPK (P-JNK, P-ERK, and P-p38) was also significantly increased; and the number of apoptotic cells in renal tubules was significantly increased. In addition, different studies have shown that inflammation and the MAPK signaling pathway play key roles in renal injury induced by ischemia–reperfusion (Su et al., 2019; Li et al., 2020). These results indicate that the MARK pathway is one of the key pathways in AKI. The PI3K-Akt signaling pathway is involved in the regulation of multiple cellular physiological processes by activating downstream effector molecules, which play an important role in the cell cycle, growth, and proliferation (Shi et al., 2019). A previous study observed that IRI gave rise to phosphorylation of AKT in WT mice; subsequently, they utilized the inhibitors of AKT (MK2206) to reconfirm the signaling pathways involved in apoptosis regulation in hypoxia-reoxygenated human TECs. In agreement with IRI, it was observed that the phosphorylation of AKT was dramatically reduced (Hou et al., 2021). These results are consistent with our research results, indicating that the TNF, PI3K-Akt, and MAPK signaling pathways are critical in the mechanism of AKI induced by ischemia–reperfusion.

Many genes undergo robust changes in the early stages of disease occurrence, including many TFs. TFs are the main regulatory factors in basic biological processes that can regulate the expression of multiple gene targets and form feedback loops. Transcription and post-translational mechanisms interact with their expression and function. It is well known that many TFs are strongly upregulated during the pathogenesis of AKI with variable timing, duration, and magnitude. Some of these include Fos, Stat3 (Bienaimé et al., 2016), Nfkb2 (Sanz et al., 2010), JUN, and P53 (Cippà et al., 2018). In a recent study, Piret and Mallipattu, (2020) found that in mice with unilateral ischemia–reperfusion kidney injury, Foxm1 was vigorously upregulated after 2 days and returned to baseline levels after 14 days. This is consistent with the trend observed in human AKI samples, which is consistent with the findings of our study. In mouse kidneys with fibrosis and inflammatory infiltration, sample sequencing to a depth of 30 million reads and network analysis showed that the top TFs, such as Irf1, Nfkb1, and Stat3, are important drivers of renal fibrosis progression (Wu et al., 2020). These TFs were also found to be important in IRI-AKI in our study. In addition, we also examined previously unrecognized TFs, such as Smad4, E2f1, Foxo1, Nfe2l2, and Ets1. These may be new candidate genes for future research on the regulation of the physiological and pathological processes in IRI-AKI. Furthermore, an miRNA–hub gene network was constructed in a recent study, which consisted of 287 mi-RNAs that might interact with hub genes, for example, miR-15a-5p, miR-15b-5p, miR-29b-3p, mir-144–3p, and miR-16–5p. A study of sepsis-induced AKI mechanism showed that miR-15a-5p, miR-15b-5p, and miR-16-5p were involved in the mTOR signaling pathway, and miR-16-5p and miR-29b-3p are involved in the PI3K-Akt signaling pathway (Xu et al., 2019).

Finally, we predicted that 318 drugs or molecular compounds might be involved in the regulation of hub genes which may be a potential drug to protect against IRI-AKI. Many studies have confirmed the effects of drugs and molecular compounds on AKI. For example, intravenously glutamine administration alleviates inflammation in obese mice with sepsis, thereby improving AKI, and this regimen is recommended for abdominal surgery in obese patients to reduce the risk of infection (Su et al., 2021). In coronary artery bypass surgery, taking aspirin within 24 h before surgery reduced the incidence of AKI after surgery by 36% and was independently associated (Aboul-Hassan et al., 2020). In contrast, ibuprofen, celecoxib, indomethacin, insulin, cefotaxin, and alogliptin were primarily associated with an increased incidence of AKI (Shen et al., 2021). These drugs or molecular compounds were mentioned in our study and could be potential drugs for the treatment of IRI-AKI in the future.

A number of measures were taken, such as all datasets from the same species, the same interventions, and normalization of the data before further analysis, to ensure the reliability of the study. However, this study had some limitations. First, the number of samples used for bioinformatics analysis was small, which may have reduced the accuracy of the result; therefore, large sample size experiments need to be considered in the future to confirm these findings. Second, our study was only used for bioinformatics analysis and lacked *in vitro* and *in vivo* experiments for verification. Next, we will carry out relevant animal and cell experiments and use experimental methods such as qPCR, Western blotting, and immunohistochemistry to study the functional mechanism in detail. Third, hub genes were identified in AKI mouse models instead of human specimens, which might restrict the clinical application of hub genes. Some clinical specimens should be collected to validate our conclusion, and further studies may better elucidate their clinical biological role.

## Conclusion

In summary, a comprehensive bioinformatics analysis of two IRI-AKI models was conducted, and it was found that several hub genes, such as *Jun*, *Stat3*, *Myc*, *Cdkn1a*, *Hif1a*, *Fos*, *Atf3*, *Mdm2*, *Egr1*, and *Ddit3*, as well as the MAPK, PI3K-Akt, and TNF signaling pathways, may play a pivotal role in the physiological and pathological processes of IRI-AKI. The analysis also revealed some TFs, mi-RNAs, and some drugs that may regulate hub genes. The findings of this study may provide potential therapeutic targets and a deeper understanding of the genetic mechanisms underlying IRI-AKI.

## Data Availability

The datasets presented in this study can be found in online repositories. The names of the repository/repositories and accession number(s) can be found in the article/Supplementary Material.
